# 1753. Impact of a Pharmacist-Driven Home Hospital Antimicrobial Stewardship Program Pilot within a Large Community Health System

**DOI:** 10.1093/ofid/ofac492.1383

**Published:** 2022-12-15

**Authors:** Van Nguyen, Michael Wankum, Krista Gens, Elizabeth B Hirsch

**Affiliations:** Abbott Northwestern Hospital, Portland, Oregon; Abbott Northwestern Hospital, Portland, Oregon; Abbott Northwestern Hospital, Portland, Oregon; University of Minnesota College of Pharmacy, Minneapolis, MN

## Abstract

**Background:**

Home Hospital (HH) is a unique and rapidly expanding care model that allows patients to receive medical therapy and monitoring through telehealth communication and nursing visits, and there are currently no published studies evaluating antimicrobial stewardship interventions in the HH setting. The goal of this study is to evaluate the impact of a pharmacist-driven antimicrobial stewardship pilot for the HH program.

**Methods:**

This was a pre-post quasi-experimental study of adult patients enrolled in HH program between January through March in 2021 (control cohort) and in 2022 (intervention cohort), who received antibiotics (oral/intravenous) during their HH admission. Patients on long-term prophylactic antimicrobials, antifungals, external antimicrobials, or mycobacterial treatment were excluded. The antimicrobial stewardship pharmacist performed prospective audit and feedback and provided recommendations to clinicians through the electronic medical record. The primary endpoint was antibiotic use (days of therapy per 1000 patient-days). Secondary endpoints included broad-spectrum antibiotic usage; appropriateness of antibiotic indication, dosing, and duration; compliance with the institution’s outpatient antibiotic reference guide or outpatient intravenous antibiotic therapy (OPAT) monitoring; treatment failure; antibiotic-associated adverse effects; and cost of antibiotic therapy.

**Results:**

The study included 73 and 127 patients in the control and intervention group, respectively (Figure 1). On average, the pharmacist reviewed 8 eligible patients/day. Interventions were generally well received by HH providers (Figure 2). There was no significant difference in the primary outcome. More inappropriate antibiotic indication was identified in the intervention group (46 [36%] vs. 15 [19%], p=0.01), associated with post-surgical infection prophylaxis after orthopedic procedures (Figure 3). Other secondary outcomes did not vary significantly between the groups.

**Figure 1:**
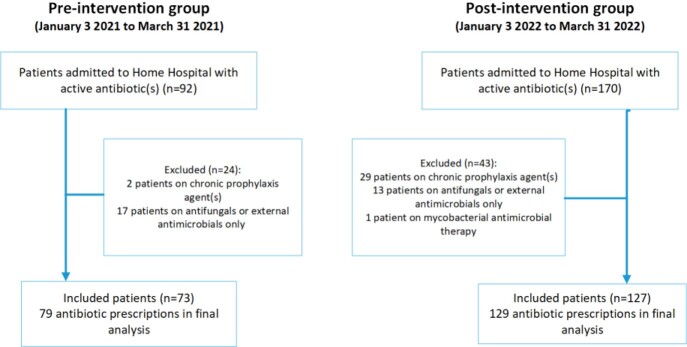
Study inclusion flowchart

**Figure 2:**
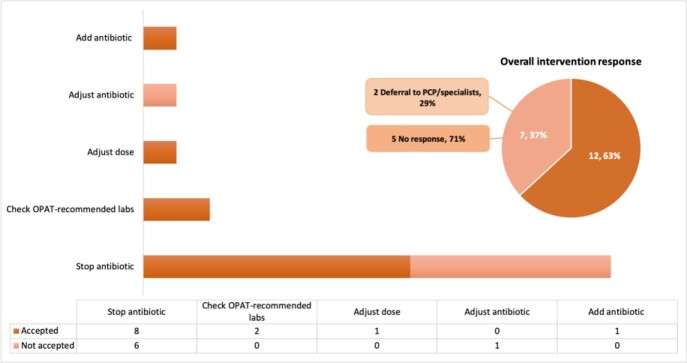
Intervention breakdown

**Figure 3:**
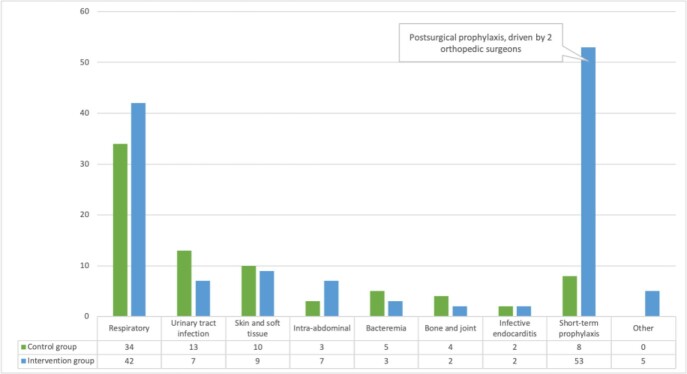
Infection types

**Conclusion:**

The pilot allows for better understanding of outpatient and HH antibiotic prescribing practices to provide targeted interventions, and suggests the need for additional antimicrobial stewardship involvement to optimize antibiotic therapy in this novel care setting.

**Disclosures:**

**Elizabeth B. Hirsch, PharmD, FCCP, FIDSA**, Melinta: Advisor/Consultant|MeMed: Advisor/Consultant|Merck: Advisor/Consultant|Merck: Grant/Research Support.

